# Emergent *Candida* Species on Healthcare Surfaces: Abiotic Reservoirs as a Source of Invasive Candidiasis

**DOI:** 10.3390/microorganisms14020367

**Published:** 2026-02-04

**Authors:** Iker De-la-Pinta, Cristina Marcos-Arias, Elena Sevillano, Elena Eraso, Guillermo Quindós

**Affiliations:** 1 Department of Immunology, Microbiology and Parasitology, Faculty of Medicine and Nursing, University of the Basque Country (UPV/EHU), 48940 Leioa, Bizkaia, Spain; iker.delapinta@ehu.eus (I.D.-l.-P.); cristina.marcos@ehu.eus (C.M.-A.); elena.sevillano@ehu.eus (E.S.); elena.eraso@ehu.eus (E.E.); 2 Instituto de Investigación Sanitaria Biobizkaia, 48903 Barakaldo, Bizkaia, Spain

**Keywords:** *Candida albicans*, *Candida (Candidozyma) auris*, *Candida parapsilosis*, dry surface biofilm, environmental persistence, resilience, healthcare-associated infections, disinfection, fomites, surfaces

## Abstract

The aetiology of invasive candidiasis is undergoing substantial changes; traditionally, these mycoses have been considered to originate from endogenous reservoirs; however, the increasing prevalence of non-*Candida albicans* species, such as *Candida parapsilosis* and *Candida auris* (also named *Candidozyma auris*), is a cause of concern as they demonstrate significant exogenous transmission. This challenges the long-standing paradigm of endogenous origin in hospital settings. Unlike previous reviews primarily focused on clinical epidemiology, this work adopts a multidisciplinary perspective combining microbiological evidence with biomaterials science. We analyse how surface roughness, hydrophobicity, and polymer composition within the hospital “plastisphere” influence *Candida* adhesion and the formation of dry surface biofilms (DSBs). In this specific context, in contrast to *C. albicans*, primarily associated with mucosal colonisation, *C. auris* and *C. parapsilosis* exhibit distinctive adaptations that promote survival in healthcare environments, including pronounced cell surface hydrophobicity and the capacity to form dense cellular aggregates, which facilitate prolonged adherence to synthetic polymers used in medical devices. We also explore the biological mechanisms underlying this resilience, with particular emphasis on the development of dry surface biofilms and viable but non-culturable states. These phenotypic traits confer tolerance to desiccation and resistance to conventional disinfectants, raising concerns that standard hygiene and decontamination protocols may be inadequate to prevent transmission. Understanding these mechanisms is essential for designing effective infection control strategies and mitigating the risk of invasive disease caused by these highly persistent species.

## 1. Introduction

Healthcare-associated infections (HAIs) remain one of the most urgent challenges in global public health, contributing substantially to morbidity, mortality, and rising healthcare costs. Contaminated surfaces in healthcare facilities play a critical role in the transmission of nosocomial pathogens, thereby driving HAIs. Transmission can occur directly from high-touch surfaces near patients or indirectly via healthcare personnel and visitors, and the risk is influenced by the level of contamination, infectious dose, pathogen virulence, hygiene practices, and patient vulnerability. Although exogenous transmission accounts for only 5–20% of HAIs in Europe, contaminated surfaces remain an important source of dissemination, primarily through hand contact. Historically infection control strategies have primarily focused on bacterial pathogens [1]; however, there has been a marked increase in invasive mycoses in recent years [2,3]. Among these, *Candida* has emerged as the leading cause of fungal HAIs and candidemia currently ranks as the fourth most common cause of nosocomial bloodstream infections, with attributable mortality rates of 30–60% despite antifungal therapy [4,5].

In 2022, the World Health Organization underscored the seriousness of this threat by publishing for the first time the Fungal Priority Pathogens List, in which *Candida albicans* and *Candida auris* (also named *Candidozyma auris*) were designated as “Critical Priority” pathogens, alongside *Cryptococcus neoformans* and *Aspergillus fumigatus* [3]. This classification reflects a growing crisis driven by two converging factors: increasing antifungal resistance and a growing population of susceptible patients, including individuals with indwelling medical devices, immunosuppression, or prolonged stays in Intensive Care Unit (ICU) [6]. While *C. albicans* remains prevalent and largely susceptible to antifungal agents, the aetiology is increasingly shifting toward non- *albicans* (NAC) *Candida* species [7]. Isolates of *Candida glabrata* (also named *Nakaseomyces glabratus*) and *Candida parapsilosis* are becoming more frequent. Recent taxonomic revisions within these genera, largely driven by genomic analyses, refine the evolutionary relationships of these fungi [8]. While these changes do not currently modify clinical management or antifungal treatment strategies, they are relevant for accurate laboratory identification, particularly when using methodologies such as MALDI-TOF MS. Such revisions may initially create uncertainty, yet they enhance our understanding of pathogenicity and virulence variation, thereby informing future diagnostic and therapeutic approaches. However, the most significant epidemiological shift beyond these taxonomic considerations has been the emergence of *C. auris* in 2009, characterised by its rapid global spread [9,10].

*C. auris* exhibits a unique multidrug-resistance (MDR) profile, with some isolates demonstrating resistance to all three major classes of antifungal agents (polyenes, azoles, and echinocandins). Furthermore, this pathogen has the exceptional ability to cause outbreaks in healthcare facilities, displaying characteristics that are more common among MDR bacteria (e.g., Methicillin Resistant *Staphylococcus aureus*—MRSA—or *Acinetobacter baumannii* complex) than among most fungi [11,12]. This behaviour suggests that the conventional infection control models, which are primarily designed for bacterial containment or endogenous fungal translocation, may prove ineffective.

Until recently, invasive candidiasis has been mainly understood as an endogenous infection in which *Candida*, as a commensal organism of the gastrointestinal tract, would translocate across mucosal barriers into the bloodstream in immunocompromised individuals or when the microbiome is disrupted by broad-spectrum antibiotics (dysbiosis). However, the growing recognition of a potential paradigm shift has led us to reconsider the origin of invasive candidiasis, shifting the focus from endogenous sources to exogenous transmission via environmental reservoirs caused by emerging species, such as *C. auris* and *C. parapsilosis*. Unlike *C. albicans*, which survival on dry surfaces is limited, these emerging species display distinct adaptive traits that enable them to persist on abiotic materials for weeks or even months [13,14].

In hospital environments, inanimate surfaces (fomites) such as bed rails, floors, call buttons and complex medical equipment act as secondary reservoirs for these pathogens [15]. Environmental research studies have detected viable *Candida* from high-touch surfaces in patient rooms, with *C. auris* showing remarkable ability to colonise both dry and moist surfaces, including sink drains and floors, thereby establishing persistent environmental niches [16,17]. This ability to persist is an active, biologically mediated process driven by defined survival mechanisms. *C. auris* and *C. parapsilosis* are typically yeast-like and do not display the complex hyphal architecture seen in *C. albicans* biofilms [18]; however, they produce a protective extracellular matrix which differentiates the sessile lifestyle from that of planktonic cells [19]. In contrast to classical hydrated biofilms described in catheters or aqueous systems, recent research has introduced the concept of the dry surface biofilm (DSB), a specialised biofilm phenotype adapted to dry hospital environments, which develops at the solid-air interface and is characterised by low moisture availability but high concentrations of extracellular polymeric substances [20]. These structures confer extreme tolerance to desiccation and chemical disinfection. Furthermore, *Candida* cells exposed to extreme stress can enter a “viable but non-culturable” (VBNC) state, remaining metabolically active and pathogenic while evading detection by standard culture-based methods [21,22].

Material properties such as surface roughness, hydrophobicity, and porosity also play a pivotal role in colonisation. For instance, synthetic polymers and plastics (ubiquitous in the hospital “plastisphere”) facilitate the attachment of hydrophobic yeast cells more effectively than natural fibres [23,24,25]. This environmental resilience promotes cross-transmission via the healthcare workers’ hands or shared equipment, perpetuating outbreaks even in the absence of an infected index patient [26].

Despite the increasing threat of nosocomial mycoses, the current literature reveals a significant gap in linking fungal physiology with the abiotic hospital environment. The existing reviews largely focus on clinical management or treat environmental persistence as static; this overlooks the fact that physicochemical material properties (such as roughness, hydrophobicity, and porosity), actively influence colonisation. Moreover, infection control guidelines often rely on planktonic susceptibility data, which fails to reflect the complexity of DSB and VBNC states, resulting in disinfection protocols that may be ineffective against these resilient phenotypes.

The primary objective of this review is therefore to bridge the gap between material science with clinical microbiology, consolidating the paradigm shift from endogenous candidiasis to exogenous transmission for these emerging species. Specifically, this work compiles the available evidence on how material-pathogen interactions shape *Candida* survival kinetics and critically evaluates disinfectant efficacy against DSB. By integrating these insights, we propose a practical, evidence-based framework for environmental hygiene that targets the specific challenges posed by emerging pathogens such as *C. auris* and *C. parapsilosis* on diverse hospital surfaces.

## 2. Materials and Methods

To assess the environmental persistence and colonisation potential of *Candida* species on hospital surfaces, a systematic literature search was performed using the Web of Science Core Collection on 6 December 2025. The search was limited to publications from January 2015 up to 6 December 2025 to capture the most recent evidence on emerging pathogens and novel surface interactions. The strategy employed three Boolean logic blocks to ensure high specificity:

TITLE: (“Candida” OR “albicans” OR “auris” OR “parapsilosis” OR “glabrata” OR “glabratus” OR “krusei” OR “kudriavzevii” OR yeast).

AND

TOPIC: (“Persistence” OR “Survival” OR “Viability” OR “resistance” OR “Dry Surface Biofilm” OR “Environmental stability” OR “Environmental persistence” OR “Surface contamination”).

AND

TOPIC: (“Abiotic surface*” OR “inanimate surface*” OR “fomite*” OR “hospital surface*” OR “Healthcare surface” OR “Environmental surface” OR “Ward furnishing” OR “device*” OR “Medical equipment” OR “Plastic*” OR “Steel” OR “Linen”).

This systematic review adheres to the PRISMA 2020 statement. The protocol was not registered. All retrieved records were imported into RefWorks, and after removing duplicates, titles and abstracts were independently screened by two reviewers against the eligibility criteria.

Given that the included studies were in vitro experimental research studies with substantial methodological heterogeneity, standard risk of bias tools designed for clinical research were not applicable. Instead, we performed a qualitative assessment of methodological quality, ensuring that only studies with clearly described protocols, appropriate controls, and reproducible outcomes were included.

## 3. Results

**Figure 1:**
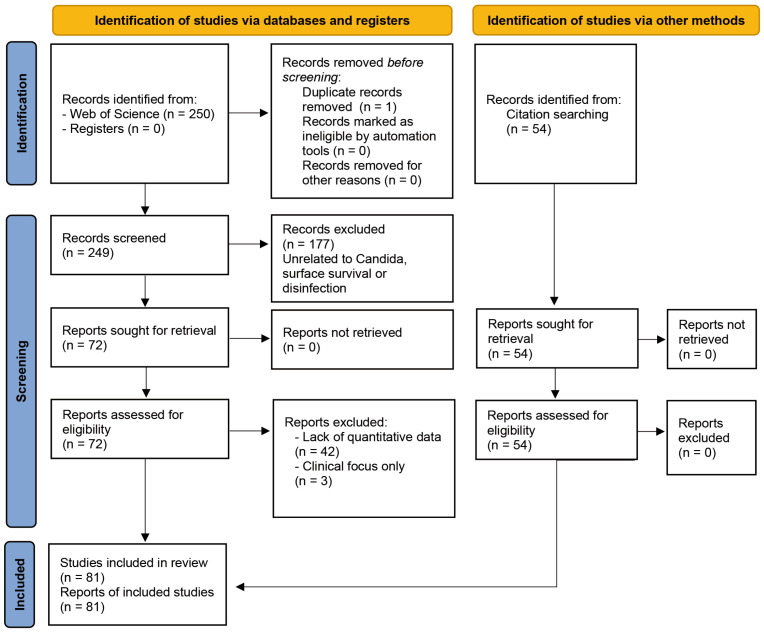
PRISMA2020 flow chart.

The systematic search in Web of Science™ Core Collection, conducted up to December 2025, initially retrieved 250 records (Figure 1). After removing one duplicate, a hierarchical screening process was carried out on 249 unique records with titles and abstracts being assessed independently by two reviewers. This first phase resulted in the exclusion of 177 records that did not meet the predefined inclusion criteria (e.g., topics unrelated to *Candida* species, surface survival, or disinfection). Consequently, 72 reports were sought for retrieval and assessed for eligibility at the full-text level. During this phase, 45 articles were excluded due to a lack of quantitative data on surface survival or disinfection (*n* = 42), or an exclusively clinical focus without environmental sampling (*n* = 3).

Ultimately, 27 primary research articles specifically focused on the survival or disinfection of *Candida* on surfaces were identified through this database search. To further enrich the review, a citation searching of the bibliographies of retrieved articles and review was performed and led to the identification of 54 additional relevant studies in PubMed that aligned the thematic scope. These supplementary references, which were all assessed as eligible, comprised 12 additional primary studies focused on candidiasis surveillance and tracking across Europe and the USA, and 42 contextual articles; which included reviews and studies addressing physicochemical factors such as surface roughness and hydrophobicity that influence *Candida* colonisation.

The final review included a total of 81 articles, with 27 from the database search and 54 from the citation search.

Across the selected studies, *C. auris* and *C. parapsilosis* consistently demonstrated prolonged survival on healthcare-relevant materials, with reported persistence ranging from 14 days to several months, markedly exceeding that of *C. albicans*. Experimental models (Table 1) showed that synthetic polymers, particularly polystyrene and polyvinyl chloride (PVC), favoured long-term persistence and facilitated the formation of DSB, whereas hydrophilic natural fibres tended to show a more rapid decline in viability due to desiccation. Regarding disinfection performance, the included studies frequently reported reduced efficacy of quaternary ammonium compounds (QACs) against dry-biofilm phenotypes. In contrast, oxidising agents, such as chlorine-based formulations and peracetic acid, generally achieved higher log-reductions. Nevertheless, some studies documented post-treatment regrowth or the emergence of VBNC states, indicating that even disinfectants with higher intrinsic activity may fail to achieve complete eradication under certain conditions [20,27].

## 4. Discussion

The epidemiology of invasive candidiasis has changed substantially in recent decades. Multiple studies have documented this shift and proposed hypotheses regarding its evolution and clinical significance worldwide [7,18,28,29,30,31,32,33,34]. *Candida* remains the leading cause of fungal HAIs, but the distribution of *Candida* species as aetiological agents is no longer static. Two concurrent trends are reshaping the epidemiology of candidemia and challenging traditional infection control measures: (1) a reversal in the ratio of *C. albicans* to NAC species, and (2) the emergence of MDR species that can persist in healthcare environments and cause nosocomial transmission, such as *C. auris* and clonal lineages of *C. parapsilosis*.

Until the late 1990s, *C. albicans* accounted for over 70% of candidemia episodes; however, surveillance now reveals a global, though heterogeneous, trend towards NAC predominance, driven by antifungal prophylaxis (especially azoles), ageing populations, and the increased use of invasive devices [35,36,37]. This shift is particularly evident in the US, where NAC species now dominate. According to CDC surveillance (2017–2021), NAC species were detected in 62.9% of cases, up from 49.3% in 1998–2006. *C. glabrata* is the second most prevalent species, accounting for approximately one-third of cases in states such as Maryland and California [38]. Spain shows a similar trend. The CANDIPOP study (2010–2011) [39] reported 54.6% NAC species, whereas the CANDIMAD study (2022) detected a 66.5%, largely due to the clonal spread of fluconazole-resistant *C. parapsilosis* [40]. Germany mirrors this pattern, with NAC species becoming increasingly prevalent in intensive care units, replacing *C. albicans* dominance [41,42,43]. In the UK, *C. albicans* remains the most frequent species (42%, in 2021), though NAC species increased from 50.6% (2010–2012) to 60.7% (2022–2024), with *C. glabrata* ranking second [44]. France shows relative stability, with *C. albicans* accounting for 55.6% of cases and just a slight increase in NAC species [45]. Norway has still the highest proportion of *C. albicans* (56.3% in 2023), despite NAC rising from 32.0% to 44.6%, likely reflecting conservative antifungal stewardship [46,47].

In healthcare environments, *Candida* species—including *C. albicans*, *C. glabrata*, *C. parapsilosis*, *Candida tropicalis*, *Candida orthopsilosis*, *Candida metapsilosis*, and *Clavispora lusitaniae*—are detected on dry and moist surfaces, confirming their environmental resilience. *C. parapsilosis* is the second leading cause of candidemia in African, Asian, and European Mediterranean countries, as well as in South America, South Africa, and Australia which highlights it importance. While the upward trend in prevalence of *C. glabrata* and *C. parapsilosis* is gradual, the emergence of *C. auris* has posed a distinct and urgent challenge. Since its first description in 2009, it has become an endemic healthcare-associated pathogen across continents, challenging fungal infection control measures. A silent colonisation by *C. auris* often precedes invasive disease, underscoring the need for strict surveillance [38,40,42,47]. Unlike other species, *C. auris* can persist in patients for over a year and can be transmitted through direct contact or via fomites such as medical devices and reusable equipment. Outbreaks have been linked to these contaminated surfaces which act as a constant source of infection and can initiate large-scale transmission, reflecting the extraordinary transmission capacity of *C. auris*. This species is characterised by its high virulence, the ability to invade the bloodstream and the resistance to multiple antifungals, which increases its potential to cause outbreaks. Clinically, *C. auris* exhibits almost universal fluconazole resistance and frequent MDR to azoles, polyenes, and echinocandins. Its ability to persist on surfaces in which they can replicate rapidly reinforces the need for stringent infection control measures targeting environmental reservoirs [7,18,28,29,30,31,32,33,34,48]. Similarly, clonal fluconazole-resistant *C. parapsilosis* (*ERG11*-Y132F substitution) is expanding in European hospitals which can also persist in the environment and transmit between patients, resembling MDR bacteria such as MRSA. Infection control must therefore extend beyond stewardship to prioritise environmental hygiene and transmission prevention [40,49].

### 4.1. Candida Colonisation and Infection: The Interplay Between Fungal Surface Properties and Adhesins

The colonisation of the hospital environment by *Candida* species is not a random process of stochastic deposition; rather, it is a selective phenomenon in which the physicochemical properties of abiotic surfaces act as environmental filters, favouring species with specific cell wall traits [50,51]. While *C. albicans* has historically served as the model organism for studying adhesion via receptor-ligand interactions on host tissues [52,53], the emergence of *C. parapsilosis* and *C. auris* calls for a re-evaluation of adhesion mechanisms on inert substrates [10,54]. The success of these emerging *Candida* species is partly attributable to their ability to exploit the thermodynamic properties of plastic polymers used in the manufacture of medical devices, as well as the micro-topographical imperfections introduced by modern industrial production processes [50,55].

A key determinant of the initial attachment of fungal cells to abiotic surfaces is cell surface hydrophobicity [24,56]. Although adhesion is often described in thermodynamic terms where hydrophobic cells displace interfacial water in order to interact preferentially with hydrophobic substrates such as silicone or polystyrene, this apparent physical phenomenon is, in fact, biologically determined by the composition and organisation of the fungal cell wall [24,57]. The expression of cell wall proteins, particularly members of the agglutinin-like sequence (Als) family, mediates these interactions at the molecular level [52,58].

Although Als proteins are classically associated with adhesion to host tissues, members of this family along with other cell wall-anchored adhesins also contribute to attachment to abiotic surfaces and medical devices [58,59]. In this context, cell surface hydrophobicity plays a central role in *Candida* adhesion to inert materials. *C. parapsilosis* exhibits higher intrinsic hydrophobicity than *C. albicans*, an attribute associated with enhanced adhesion to hydrophobic substrates and prolonged survival on synthetic hospital materials [60,61]. This physicochemical advantage likely contributes to the strong association of *C. parapsilosis* with catheter-related infections, where adhesion to polymeric devices represents a critical pathogenic step.

Unlike *C. parapsilosis*, which has a predominantly hydrophobic strategy, *C. auris* has developed a multimodal adhesion strategy that likely underpins its exceptional environmental persistence. Recent studies have challenged the conventional Als-based framework by identifying lineage-specific adhesins, such as Scf1 (surface colonisation factor 1), which operate through mechanisms distinct from classical hydrophobic forces. As demonstrated by Santana et al., Scf1 promotes surface binding via domains enriched in cationic residues, suggesting electrostatic interactions with negatively charged substrates including human skin and certain medical polymers [62]. This electrostatic mechanism, acting in association with the hydrophobic activity of conserved adhesins, such as Iff4109, confers upon *C. auris* a hybrid adhesive phenotype; this enables it to colonise a broader spectrum of abiotic surfaces than species that rely solely on hydrophobic interactions [62,63].

A defining biophysical characteristic of epidemic *C. auris* clades is their aggregative phenotype. Unlike the predominantly planktonic growth of *C. albicans*, *C. auris* cells frequently fail to separate after cytokinesis, forming large, multilayered aggregates [64,65]. This aggregation constitutes an adaptive survival strategy mediated by Als/Iff-family adhesins, including lineage-specific proteins such as Iff4109 and Scf1 [62,63]. These cellular clusters exhibit enhanced resistance to shear forces and disinfectants, thereby effectively shielding inner cells from environmental stressors [66]. Consequently, what appears macroscopically as a single colony-forming unit may actually represent a highly organised, drug-tolerant consortium capable of initiating rapid biofilm formation upon host contact.

While surface chemistry governs initial attachment, surface topography determines biofilm retention and resistance to removal. Recent experimental data have confirmed a significant positive correlation between increasing roughness and *Candida* adhesion across diverse polymeric materials [67,68]. These findings support the established consensus of an *Ra* (roughness average) threshold of 0.2 µm, below which microbial retention is theoretically minimised, although not entirely prevented [69]. Furthermore, these results reinforce the broader concept that surface irregularities promote microbial retention by providing protected micro-niches that reduce the effectiveness of shear forces and surface decontamination, a mechanism widely discussed in models of biofilm formation on biomedical materials. This “shelter effect” is particularly relevant to the epidemiology of *C. auris* and *C. parapsilosis* as these species, unlike *C. albicans*, rely heavily on environmental reservoirs to sustain nosocomial transmission [66,70,71].

Hospital textiles represent an underappreciated but critical environmental reservoir for *Candida* transmission. Neely and Orloff demonstrated that *Candida* viability on fabrics is material-dependent, with synthetic fibres such as polyester supporting fungal survival for significantly longer periods than natural fibres like cotton [60]. Subsequent studies have confirmed that low moisture absorption and reduced disinfectant penetration within tightly woven synthetic textiles contribute to prolonged *Candida* persistence [72]. This phenomenon is consistent with the hydrophobic phenotype of *C. parapsilosis* and the aggregative behaviour of *C. auris*. It raises the possibility that the growing reliance on synthetic healthcare textiles may unintentionally enhance the environmental persistence of these pathogens. As a result, healthcare workers’ garments may function as mobile reservoirs, facilitating indirect transmission within the hospital ecosystem [60,71].

### 4.2. Dry Surface Biofilms (DSBs)

The classical conceptualisation of fungal biofilms has been largely derived from hydrated environments, such as intravascular catheters or mucosal surfaces, where microbial communities develop at solid–liquid interfaces with continuous nutrient availability [18]. However, this model is insufficient to explain the persistence dynamics of emerging *Candida* species in the hospital environment, where moisture is intermittent and desiccation is a constant stressor. Recent evidence requires the recognition of the DSB as a distinct phenotype, adapted to the solid-air interface and representing the primary mode of existence for pathogens like *C. auris* and *C. parapsilosis* on fomites [20]. Unlike standard *Candida* hydrated biofilms, which are typically thick and water-rich structures dominated by hyphal elements (as in *C. albicans*), *Candida* DSBs consist of densely packed yeast cells embedded in a concentrated extracellular polymeric substance matrix with minimal free water. This architecture is not merely a cluster of desiccated cells but a biologically active community formed through cycles of accumulation, hydration, and evaporation [20].

Experimental models utilising cyclic hydration and desiccation protocols have demonstrated that *C. auris* can develop mature DSBs over 12 to 14 days on clinically relevant substrates, such as stainless steel and polymers [21,27]. Structurally, these biofilms display multi-layered architectures where the extracellular polymeric substance matrix acts as a hydrogel, retaining residual moisture and stabilising cell-to-cell adhesion. This matrix is functionally critical; studies comparing aggregating and non-aggregating phenotypes of *C. auris* show that aggregating isolates, which naturally produce more matrix and exhibit stronger adhesion, survive desiccation far better than single-celled isolates, maintaining viability for weeks where *C. albicans* fails to persist [21,71]. Furthermore, this persistence is amplified in physiological matrices relevant to the skin, such as synthetic sweat, where *C. auris* forms high-burden biofilms up to ten times denser than those of *C. albicans* [71].

A defining feature of DSBs is their extreme recalcitrance to environmental stressors and chemical disinfection, a feature that goes beyond simple physical shielding. Recent transcriptomic profiling of *C. auris* DSBs has provided the first mechanistic insights into this phenotype. Compared to planktonic cells, *C. auris* within DSBs significantly upregulates genes associated with efflux pumps (specifically ATP-binding cassette transporters *CDR1* and *CDR4*) and iron acquisition pathways (siderophore transporters, such as *SIT1*) [27]. This suggests that the DSB state actively primes the fungal cell for detoxification and metabolic survival in nutrient-poor environments.

This molecular reprogramming translates into observable tolerance. While planktonic *C. auris* is rapidly killed by standard hospital disinfectants, DSBs show reduced susceptibility. For instance, DSBs of *C. auris* have demonstrated the ability to regrow after treatment with 1000 ppm of sodium hypochlorite (NaOCl) and hydrogen peroxide (H_2_O_2_), agents typically considered effective [20,27]. Moreover, repeated exposure to sub-lethal concentrations of disinfectants like benzalkonium chloride has been shown to induce adaptive low-level resistance in *C. auris*, characterised by efflux pump activation and modulation of membrane ergosterol content [16].

The hazard posed by DSBs is further exacerbated by the limitation of standard culture-based surveillance. Desiccation stress within the biofilm drives a subpopulation of cells into a VBNC state. Welsh et al. demonstrated that although cultivability of *C. auris* on plastic surfaces declines within two weeks, metabolic activity—assessed by esterase activity—persists for at least 28 days, significantly longer than *C. parapsilosis* [14]. These VBNC cells retain pathogenic potential; recent data indicates that *C. auris* recovered from plastic marine pollution after 30 days of environmental exposure maintains full virulence in *Galleria mellonella* models [25]. This implies that hospital surfaces testing “culture-negative” may still harbour metabolically active biofilms capable of reactivation and transmission.

The interaction between DSBs and surface materials is pivotal. The modern hospital environment, rich in polymers, creates a kind of “plastisphere” that favours the adhesion and persistence of hydrophobic and aggregative *Candida* species. *C. auris* has been shown to persist longer and transfer more efficiently from plastic and latex surfaces than from glass or steel [25,73]. Furthermore, the transferability of *C. auris* from DSBs to other surfaces remains high even after disinfection protocols that successfully reduce viable counts, highlighting that “killing” does not necessarily equate to “removing” the risk of transmission [20]. Thus, the DSB represents a sophisticated survival strategy that allows *Candida* to survive independently of the host. By transforming inert fomites into active, resistant reservoirs, DSBs challenge current infection control frameworks, which often underestimate the resilience of surface-associated populations. Effective eradication requires protocols that not only kill the organism but physically disrupt the DSB matrix and account for the VBNC state.

### 4.3. Environmental Survival and Persistence of Candida

The persistence of *Candida* species on abiotic surfaces is not uniform; it is a multifactorial phenomenon influenced by fungal species, the physicochemical properties of the surface, and environmental conditions such as relative humidity and organic soiling [13,74]. As detailed in Table 1, experimental models indicate that while *C. albicans* exhibits limited resilience to desiccation, emerging NAC species have evolved mechanisms to maintain viability for weeks or even months, establishing environmental reservoirs analogous to those described for vegetative bacteria like *Acinetobacter* or *Enterococcus*.

**Table 1:** Summary of experimental studies on the survival and persistence of *Candida* on abiotic surfaces.

Authors (Year) [Reference]	Assay				Comments
	Species	Inoculum	Material (Surface)	Assay Time	
Rangel-Frausto et al. (1994) [75]	*C. albicans*	100 µL of 5 × 10^5^ CFU/mL (McFarland 1.0 density suspension)	Sterile plastic lids	24 h	*C. albicans* persists on inanimate surfaces for >24 h. Transmission rates from plastic to hands remain high (90%) even 1 h post-inoculation, underscoring significant cross-infection potential.
Neely & Orloff (2001) [60]	*C. albicans*, *C. tropicalis*, *C. krusei*, *C. parapsilosis*	10 µL aliquot containing 10^4^ to 10^5^ CFU	Fabrics: 100% cotton, cotton terry, cotton-polyester blends, polyester, spandex/nylon. Plastics: Polyethylene and polyurethane.	Up to 31 days	*C. parapsilosis* exhibits significantly prolonged survival (median 30 days) compared to other species (~4 days). Persistence is enhanced on synthetic materials (polyester, plastic) versus natural fibres, implicating these substrates as critical transmission fomites.
Traoré et al. (2002) [13]	*C. albicans*, *C. parapsilosis*	10 µL of 10^9^ CFU/mL suspension (containing a soil load)	Non-porous: Glass vials and stainless-steel discs. Porous: 100% cotton and cotton/polyester blend fabrics. Biotic: Human skin (finger pads).	14 days (surfaces) 60 min (hands)	*C. parapsilosis* outlasts *C. albicans* on non-porous surfaces (14 vs. 3 days). Conversely, fabrics facilitate *C. albicans* survival (up to 14 days), likely due to moisture retention. On hands, viability is maintained for up to 60 min.
Weaver et al. (2010) [76]	*C. albicans*	20 µL drop containing 2.9 × 10^7^ cells	Copper (C11000) and aluminium coupons	24 days (576 h)	While *C. albicans* persists on aluminium (>24 days), copper surfaces exhibit rapid fungicidal activity (eradication within 24 h), mediated by germination inhibition and metabolic disruption.
Welsh et al. (2017) [14]	*C. auris*, *C. parapsilosis*	10 µL of 5 × 10^6^ cells/mL suspended in artificial test soil (ATS)	PVC acrylic alloy plastic coupons (Kydex-T)	28 days	*C. auris* remains culturable for ≥14 days and metabolically active (esterase activity) for 28 days, indicating entry into a VBNC state. Recovery from complex communities necessitates high-salinity/high-temperature enrichment to prevent false negatives.
Piedrahita et al. (2017) [17]	*C. auris*, *C. albicans*, *C. glabrata*, *C. parapsilosis*	10 µL containing 10^6^ CFU suspended in phosphate-buffered saline (PBS)	Non-porous steel discs and moist non-nutrient agar sections	7 days (sampled at 2 h, 1, 2, and 7 days)	Examined species persist for 7 days on both dry and moist surfaces. However, environmental sampling yields significantly higher recovery from moist sites (e.g., sinks), identifying them as primary transmission reservoirs.
Biswal et al. (2017) [77]	*C. auris*	100 µL of 10^6^ CFU/mL	Hospital linen and blankets	Up to 10 days	*C. auris* survives on dry hospital linens for up to 7 days, confirming the role of textiles in facilitating rapid pathogen propagation within ICUs.
Short et al. (2019) [21]	*C. auris* (aggregating and single-cell phenotypes), *C. glabrata*, *C. parapsilosis*	Standardised cell suspension of 1 × 10^8^ cells/mL	Thermanox™ coverslips	14 days	The aggregative phenotype significantly enhances survival (>14 days) and tolerance to sodium hypochlorite (1000 ppm) compared to single-celled isolates. Resilience correlates with the upregulation of biofilm-associated genes (adhesion, matrix, efflux).
Ledwoch & Maillard (2019) [20]	*C. auris* (DSM 21092)	1 mL suspension of 1 × 10^6^ CFU/mL (in organic load)	Stainless steel discs (AISI 430)	12 days (alternating hydration/desiccation cycles)	*C. auris* develops resilient Dry Surface Biofilms (DSBs) on stainless steel via sequential hydration/desiccation cycles (12 days), rendering them recalcitrant to standard elimination protocols.
Horton et al. (2020) [71]	*C. auris*, *C. albicans*	Plastic: 200 µL of 1 × 10^6^ cells/mL Porcine skin: 10 µL of 1 × 10^7^ cells/mL	Polystyrene microtiter plates, coverslips, and ex vivo porcine skin	Up to 14 days	In synthetic sweat, *C. auris* forms high-burden biofilms (10-fold biomass vs *C. albicans*) resistant to desiccation. Efficient proliferation in multilayer biofilms on porcine skin elucidates its propensity for cutaneous colonisation and nosocomial spread.
Khodadadi et al. (2022) [73]	*C. auris*, *C. albicans*, *C. parapsilosis*, *C. glabrata*	Solutions of 10^4^ CFU/mL	Sheets of cotton textile, polystyrene, paper, aluminium, glass, latex, and dried Sabouraud dextrose agar	Up to 120 days (sampled at 1, 2, 7, 14, 30, 45, 60, and 120 days)	Latex and polystyrene act as high-risk matrices supporting persistence for up to 30 days. Organic soiling (e.g., dried nutrient residues) extends survival to >120 days, significantly amplifying environmental persistence.
Dire et al. (2023) [16]	*C. auris* (clinical isolates), *C. albicans*	100 µL of 10^6^ CFU/mL	Polypropylene plastic, glass, timber wood, cotton fabric, and stainless steel (grade 304)	21 days (3 weeks)	*C. auris* survives > 21 days on diverse surfaces, with wet wood uniquely promoting active growth. Intermittent exposure to sub-lethal disinfectant concentrations induces adaptive resistance via efflux pump activation.
Akinbobola et al. (2024) [25]	*C. auris* (aggregating and non-aggregating phenotypes)	Surfaces: 300 µL suspension (in sterile human faecal material) Microbeads: 1 × 10^6^ CFU/mL	HDPE sheets, polyethylene microbeads, glass microbeads, and glass slides	30 days	Plastic contaminants serve as a novel environmental reservoir, supporting *C. auris* survival for >30 days in aquatic settings. Transfer from microplastics to beach sand occurs, particularly under moist conditions, posing an emerging public health risk.
Ware et al. (2025) [27]	*C. auris*	1 × 10^6^ cells/mL standardised in RPMI	Polystyrene microtiter plates and Thermanox coverslips	12 days (3 cycles of 48 h hydrated/48 h dry conditions)	DSBs develop adaptive tolerance to sodium hypochlorite through repeated exposure cycles. Transcriptomics reveals that upregulation of efflux pumps (*CDR1*, *CDR4*) and iron acquisition pathways drives persistence, compromising the efficacy of standard disinfection against mature biofilms.

CFU: colony forming units; DSB: dry surface biofilms; HDPE: high-density polyethylene; PVC: polyvinyl chloride; VBNC: viable but non-culturable.

Historical data established distinct survival kinetics among *Candida* species. Neely and Orloff demonstrated that clinical isolates of *C. parapsilosis* persisted for periods exceeding 30 days on standard hospital fabrics and plastics, whereas *C. albicans* and *C. tropicalis* were usually no longer recoverable after 1–4 days [60]. This species-specific dichotomy was corroborated by Traoré et al. who reported that *C. parapsilosis* remained viable for 14 days on glass and stainless steel, while *C. albicans* cell populations declined below detection limits within three days on the same non-porous substrates [13].

The emergence of *C. auris* has led to the presence of a pathogen with environmental tenacity comparable or even higher to that of *C. parapsilosis*. Piedrahita et al. compared the survival of eight *C. auris* isolates with those from other species on steel discs, finding that the percentage of *C. auris* isolates that were able to persist at seven days was greater than those of *C. albicans* but similar to *C. parapsilosis* and *C. glabrata* [17]. However, survival is phenotype-dependent: Short et al. observed that aggregative isolates of *C. auris* maintained viability on plastic for 14 days, whereas single-cell isolates showed significantly reduced recovery, indicating that cellular aggregation confers a protective advantage against desiccation stress [21].

The interaction between the *Candida* cell wall and surface topography markedly influences persistence. Polymeric substrates promote prolonged survival: Khodadadi et al. demonstrated that latex and polystyrene surfaces supported viable populations of *C. auris* and *C. parapsilosis* for up to 30 days at ambient temperature, significantly exceeding survival on glass or aluminium [73]. Similarly, Akinbobola et al. recovered viable *C. auris* from high-density polyethylene (HDPE) after 30 days of exposure to aquatic environments [25]. Regarding hospital textiles, synthetic fibres (polyester) have been shown to support fungal survival longer than natural fibres (cotton), likely due to the hydrophilic nature of cotton accelerating desiccation [60]. Conversely, the surface material itself can also exert fungicidal activity. Weaver et al. (2010) reported the complete killing of *C. albicans* within 24 h on copper coupons (C11000), accompanied by the inhibition of germination, whereas populations on aluminium remained viable for over 24 days [76]. In contrast to the inhibitory effect of copper, porous organic substrates may support proliferation; for instance, Dire et al. reported that *C. auris* not only survived but exhibited a 1-log increase on wet wood surfaces over 21 days, presumably utilising lignocellulosic components as a nutrient source [16].

Standard laboratory assays using clean substrates may underestimate the environmental resilience of *Candida* in clinical settings, where surfaces are often contaminated with biological fluids. Rangel-Frausto et al. observed that *C. albicans* survival increased when associated with faecal material or proteinaceous matrices [75]. More recently, Horton et al. demonstrated that *C. auris* cultured in a synthetic sweat medium developed high-burden biofilms that resisted desiccation for 14 days, exhibiting a biomass tenfold greater than *C. albicans* biofilms under identical conditions [71]. Consistent with this, Khodadadi et al. observed that when *C. auris* was inoculated onto dried Sabouraud dextrose agar (simulating a nutrient-rich spill), its survival extended beyond 120 days, compared to the 30 days it shows on inert surfaces [73].

The absence of colony formation does not necessarily indicate sterility. Welsh et al. used esterase activity assays to demonstrate that although *C. auris* cultivability on plastic surfaces declined over 14 days, metabolically active cells persisted for at least 28 days [14]. This VBNC state represents a critical diagnostic gap in environmental surveillance. These cells retain pathogenic potential. Akinbobola et al. confirmed that *C. auris* recovered from plastics after 30 days of environmental exposure maintained full virulence in *G. mellonella* infection models [25]. Furthermore, Rangel-Frausto et al. quantified a 90% transfer rate of *C. albicans* from plastic surfaces to hands even after one hour of drying [75], confirming that these persistent populations remain an active source of exogenous transmission.

### 4.4. Strategies for Environmental Control and Disinfection

Effective decontamination of the healthcare environment constitutes the primary intervention to interrupt the exogenous transmission of *Candida*. However, current protocols often rely on bactericidal standards that do not adequately address the resilience of fungal DSB. The efficacy of environmental control strategies depends not only on the choice of the active agent but also on the formulation, contact time, and the mechanical rigour of the application [20]. In the light of the methodological variability and the absence of widely standardised environmental decontamination protocols across studies, a rigorous comparison of individual disinfectants and dose–response parameters falls outside the remit of this review. We therefore focus on cross-cutting mechanisms, particularly DSB and polymer-dependent surface characteristics that plausibly modulate disinfection outcomes and should be addressed in future, standardised evaluations.

Quaternary ammonium compounds (QACs) remain widely used in hospitals due to their material compatibility. However, experimental evidence consistently demonstrates their inadequacy against emerging *Candida* species. Cadnum et al. reported that QAC-based disinfectants failed to achieve significant log reductions against *C. auris* on steel discs, performing significantly worse than against *C. albicans* [78]. This lack of efficacy is exacerbated in biofilm conditions. In a study assessing 12 commercial disinfectants (including liquids and wipes), Ledwoch and Maillard observed that QAC-based formulations were insufficient to reduce viability, with 58% of the tested products unable to prevent *C. auris* transfer to clean surfaces [20]. Furthermore, recent data suggest that the use of these agents may be counterproductive: Dire et al. provided mechanistic evidence that repeated exposure to sub-lethal concentrations of benzalkonium chloride induces low-level resistance in *C. auris* via the activation of efflux pumps and modulation of membrane ergosterol [16].

In contrast to QACs, oxidising agents, particularly peracetic acid (PAA) and sodium hypochlorite (NaOCl), demonstrate superior fungicidal activity against biofilms. Ledwoch and Maillard identified that products containing 3500 ppm PAA or 1000 ppm available chlorine (concentrations typically recommended for terminal cleaning or outbreak control) were the only agents capable of preventing *C. auris* biofilm regrowth and transfer [20]. Evidence regarding chlorine dioxide (ClO_2_) requires careful interpretation. While Ledwoch and Maillard observed poor performance of ClO_2_ against the resilient *C. auris*, Norville et al. recently reported rapid (>5-log10) inactivation of the pathogen [79]. However, it is important to note that the latter study assessed surface-adhered cells rather than fungal biofilms, suggesting that its ability to penetrate and eradicate the mature fungal DSB matrix may be limited compared to PAA or NaOCl. Nevertheless, the efficacy of is not absolute: Ware et al. recently demonstrated that, while planktonic cells are highly susceptible to NaOCl, mature *C. auris* DSBs develop progressive tolerance over repeated hydration-desiccation cycles. In their study, even high concentrations of NaOCl (1000 ppm) resulted in only a 2–4-log10 reduction in viable cells within mature biofilms, driven by upregulation of ATP-binding cassette (ABC) transporters [27]. These findings indicate that although oxidising agents are superior, standard protocols may fail to fully eradicate established, mature dry biofilms.

Real-world outbreak investigations underline the need for targeted, high-level disinfection protocols that combine chemical efficacy with mechanical removal. Biswal et al. documented a persistent *C. auris* outbreak in an ICU where standard phenol-based cleaning proved ineffective. Environmental sampling revealed extensive contamination of bed rails, trolleys, and medical equipment. The outbreak was controlled only after switching to a stabilised hydrogen peroxide/silver nitrate disinfectant (Ecoshield) combined with implementing a rigorous “two-bowl” cleaning method to prevent mop re-contamination [77]. This intervention highlighted that application technique is as critical as the chemical agent itself. Finally, “no-touch” automated technologies such as UV-C light have been evaluated as adjuncts to manual cleaning [12,80], but their activity is limited by shadowing and distance, reinforcing that they cannot replace meticulous manual disinfection with fungicidal agents.

## 5. Conclusions and Future Perspectives

**Figure 2:**
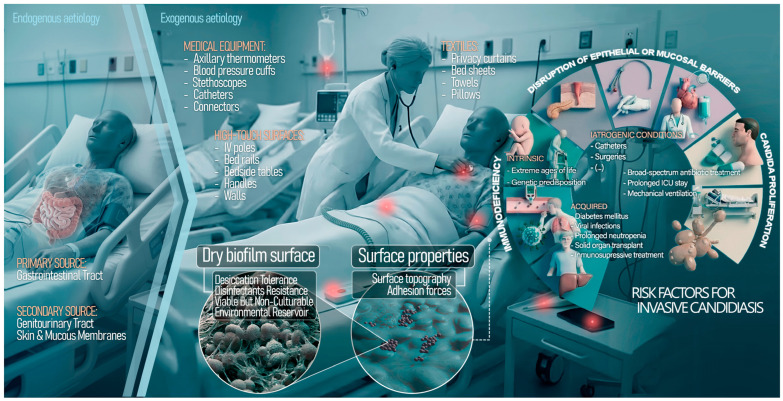
Transition from endogenous infection to exogenous transmission: the hospital environment as a reservoir in invasive candidiasis.

This review integrates evidence from clinical microbiology and materials science to shed light on the main drivers of the epidemiological shift in invasive candidiasis: the transition from an endogenous aetiology towards a model where the inanimate hospital environment serves as a significant reservoir for exogenous transmission (Figure 2). By bringing together fungal physiology and the physicochemical characteristics of hospital surfaces (e.g., roughness and hydrophobicity), this analysis identifies three factors redefining the risk profile of *Candida* species. First, the epidemiological behaviour of *C. auris* and *C. parapsilosis* more closely resembles that of MDR bacteria such as *Acinetobacter* or MRSA than that of *C. albicans*, as these yeasts are able to colonise and persist within the “plastisphere” of modern healthcare environments for extended periods. Second, this persistence is not passive but reflects an active metabolic state driven by DSB formation and the transition into a VBNC state [14,20]. Third, current infection control protocols often underestimate this threat because they rely on planktonic testing standards that fail to account for the extreme tolerance of desiccated biofilms to chemical disinfection.

Consequently, the mitigation of these environmental reservoirs requires pragmatic, evidence-based optimisation of hygiene protocols. The evidence suggests that QACs should be restricted in high-risk units, as they are often ineffective against fungal DSBs and may induce efflux-mediated tolerance in *C. auris* [16,20,78]. Instead, environmental hygiene must prioritise oxidative chemicals, such as sodium hypochlorite or PAA, applied with sufficient mechanical friction to disrupt the biofilm matrix. Furthermore, given the strong affinity of these hydrophobic *Candida* species for synthetic textiles [60,72], healthcare workers’ uniforms must be recognised as active vectors (fomites) requiring rigorous laundry and disinfection cycles, particularly during outbreaks.

Future perspectives include closing the gap between laboratory testing and clinical reality. There is an urgent need to standardise methods that mimic conditions of hospital surfaces (dryness and lack of nutrients) rather than favourable laboratory conditions. Additionally, rapid environmental screening tools capable of detecting VBNC populations are essential to guide “no-touch” decontamination interventions (e.g., UV-C or hydrogen peroxide vapour) more effectively [12]. Ultimately, effective control of *C. auris* and *C. parapsilosis* requires the recognition that hospital surfaces constitute biologically active interfaces that require the same level of antimicrobial stewardship and rigorous hygiene as that applied to patient care.

## Data Availability

No new data were created or analyzed in this study. Data sharing is not applicable to this article.

## Abbreviations

The following abbreviations are used in this manuscript:

ABC	ATP-binding cassette
Als	Agglutinin-like sequence
ATS	Artificial test soil
DSB	Dry Surface Biofilm
HAI	Healthcare-Associated Infections
HDPE	High-Density PolyEthylene
ICU	Intensive Care Unit
MDRA	MultiDrug-Resistance
MRSA	Methicillin Resistant Staphylococcus aureus
NAC	non-*Candida albicans*
PAA	Peracetic acid
PBS	Phosphate-buffered saline
PVC	Polyvinyl chloride
QAC	Quaternary Ammonium Compounds
Ra	Roughness Average
Scf1	Surface Colonisation Factor 1
VBNC	Viable But Non-Culturable
